# Aqueous *Thunbergia laurifolia* leaf extract alleviates paraquat-induced lung injury in rats by inhibiting oxidative stress and inflammation

**DOI:** 10.1186/s12906-022-03567-4

**Published:** 2022-03-22

**Authors:** Sarawoot Palipoch, Chuchard Punsawad, Phanit Koomhin, Prasit Na-Ek, Wasinee Poonsawat, Rungruedi Kimseng, Potiga Chotipong, Kingkan Bunluepuech, Gorawit Yusakul, Prasit Suwannalert

**Affiliations:** 1 School of Medicine, Walailak University, 222 Thaiburi, Thasala District, Nakhon Si Thammarat, 80161 Thailand; 2 Research Institute for Health Sciences, Walailak University, Nakhon Si Thammarat, 80160 Thailand; 3 Center of Scientific and Technological Equipment, Walailak University, Nakhon Si Thammarat, 80160 Thailand; 4 School of Medicine, Walailak University, Nakhon Si Thammarat, 80160 Thailand; 5 Research Excellence Center for Innovation and Health Product, Walailak University, Nakhon Si Thammarat, 80160 Thailand; 6 School of Pharmacy, Walailak University, Nakhon Si Thammarat, 80160 Thailand; 7 Department of Pathobiology, Faculty of Science, Mahidol University, Bangkok, 10400 Thailand

**Keywords:** Paraquat, *Thunbergia laurifolia*, Lung injury, Oxidative stress, Inflammation, Malondialdehyde, NADPH oxidase, Interleukin 1 beta, Tumor necrosis factor alpha

## Abstract

**Background:**

Paraquat (PQ) has been reported to have a high mortality rate. The major target organ of PQ poisoning is the lungs. The pathogenesis of PQ-induced lung injury involves oxidative stress and inflammation. Unfortunately, there is still no effective antidote for PQ poisoning. We hypothesized that aqueous *Thunbergia laurifolia* (TL) leaf extract is a possible antidote for PQ-induced lung injury.

**Methods:**

The total phenolic content and caffeic acid content of an aqueous extract of TL leaves were analyzed. Male Wistar rats were randomly divided into four groups (*n* = 4 per group): the control group (administered normal saline), the PQ group (administered 18 mg/kg body weight (BW) PQ dichloride subcutaneously), the PQ + TL-low-dose (LD) group (administered PQ dichloride subcutaneously and 100 mg/kg BW aqueous TL leaf extract by oral gavage) and the PQ + TL-high-dose (HD) group (administered PQ dichloride subcutaneously and 200 mg/kg BW aqueous TL leaf extract by oral gavage). Malondialdehyde (MDA) levels and lung histopathology were analyzed. In addition, the mRNA expression of NADPH oxidase (NOX), interleukin 1 beta (IL-1β), and tumor necrosis factor alpha (TNF-α) was assessed using reverse transcription-polymerase chain reaction (RT-PCR), and the protein expression of IL-1β and TNF-α was analyzed using immunohistochemistry.

**Results:**

The total phenolic content of the extract was 20.1 ± 0.39 μg gallic acid equivalents (Eq)/mg extract, and the caffeic acid content was 0.31 ± 0.01 μg/mg. The PQ group showed significantly higher MDA levels and NOX, IL-1β and TNF-α mRNA expression than the control group. Significant pathological changes, including alveolar edema, diffuse alveolar collapse, hemorrhage, leukocyte infiltration, alveolar septal thickening and vascular congestion, were observed in the PQ group compared with the control group. However, the aqueous TL leaf extract significantly attenuated the PQ-induced increases in MDA levels and NOX, IL-1β and TNF-α expressions. Moreover, the aqueous TL leaf extract ameliorated PQ-induced lung pathology.

**Conclusion:**

This study indicates that aqueous TL leaf extract can ameliorate PQ-induced lung pathology by modulating oxidative stress through inhibition of NOX and by regulating inflammation through inhibition of IL-1β and TNF-α expressions. We suggest that aqueous TL leaf extract can be used as an antidote for PQ-induced lung injury.

**Supplementary Information:**

The online version contains supplementary material available at 10.1186/s12906-022-03567-4.

## Background

Paraquat (PQ) is a bipyridylium herbicide that has been shown to be severely toxic to humans. PQ exposure, either accidental or intentional, is associated with high mortality rates. The incidence rate of PQ intoxication is at least 3.8 cases per 100,000 inhabitants annually [1]. The ingested amount of PQ is the key factor affecting the prognosis of patients [1]. The 50% lethal dose (LD_50_) in humans is approximately 35 mg/kg [2]. PQ accumulates mainly in the lungs, specifically in type I and type II pneumocytes and Clara cells, at 6–10 times the levels in plasma [3]. The lung pathology associated with PQ poisoning involves destructive and proliferative phases [4]. The destructive phase comprises alveolar epithelial swelling and fragmentation, alveolar edema and acute inflammation, while the proliferative phase involves diffuse intra-alveolar fibrosis via promotion of fibroblast infiltration into the alveolar space. In previous study, histological analysis revealed that the lungs of 11 patients who died from PQ poisoning demonstrated hemorrhage, macrophage extrusion, edema, honeycombing, fibrosis and, rarely, epithelial hyperplasia [5].

Oxidative stress, reduced antioxidant capacity and inflammation are key mechanisms of PQ-induced lung injury [6]. Oxidative stress results from imbalance between the generation and clearance of oxidants. PQ induces oxidative stress by inducing the production of large amounts of reactive oxygen species (ROS), including superoxide radicals (O_2_^•-^), hydroxyl radicals (^•^OH), and hydrogen peroxide (H_2_O_2_), via redox cycling [7]. ROS are highly reactive with cellular macromolecules such as lipids, proteins and nucleic acids, and ROS reactions cause lipid peroxidation, protein carbonylation and DNA damage. NADPH oxidase (NOX), which is widely distributed in several tissues and organs, typically catalyzes the reduction of molecular oxygen (O_2_) to produce O_2_^•-^ [8]. Notably, high levels of PQ cause ROS-mediated inflammation by enhancing the levels of pro-inflammatory cytokines such as tumor necrosis factor alpha (TNF-α) and interleukin 1 beta (IL-1β) [9].

Currently, there is still no proven antidote or effective treatment for PQ poisoning. However, novel compounds with antioxidant and/or anti-inflammatory properties have been explored, especially traditional herbal medicines, because of their efficacy and safety. A traditional Thai herb*, Thunbergia laurifolia* (TL), exhibits various biological properties, particularly curative, anti-inflammatory and antioxidant properties. TL has been widely used as a treatment agent for poisoning caused by toxic substances such as drugs, alcohol and heavy metals [10, 11]. TL aqueous extract at 25 mg/kg (orally) for 7 days showed the hepatoprotective activity against ethanol induced liver injury both in vitro and in vivo [12]. TL leaf extract at 100 mg/kg or 200 mg/kg body weight (orally) once a day can alleviate neuronal cell death and memory loss and restore via antioxidant activities in mice [10]. Wistar rats have been widely used to determine pharmacological effects of TL against toxicants [13, 14]. Therefore, the present study aimed to investigate the protective effect of an aqueous TL leaf extract against PQ-induced lung injury in male Wistar rats.

## Methods

### Preparation of aqueous extract of TL leaves

Leaves of TL were obtained from Nakhon Si Thammarat Province, Thailand (voucher specimen *Thunbergia laurifolia* AHS2008120101) and had been deposited at the Herbarium of Plant Genetic Conservation Project under The Royal Initiative of Her Royal Highness Princess Maha Chakri Sirindhorn (RSPG), Walailak University, Nakhon Si Thammarat. The leaves were washed and dried at 60 °C in an oven and ground into powder using a blender. The leaf powder (10 g) was boiled in 100 ml of distilled water for 48 min. The filtrate was lyophilized in a freeze dryer (Eyela, Tokyo, Japan) and preserved at − 80 °C until use.

### Analysis of total phenolic content

The content of phenolic compounds in aqueous extract of TL was determined using Folin-Ciocalteu reagent, which the method was described previously [15]. The working solution was made by dissolving the TL extract in water to a concentration of 2.5 mg/ml and diluting it serially. The calibration curve was produced using gallic acid (GA) solutions with concentrations ranging from 15.6 to 500 μg/mL. Sample or GA solution (GA) (20 μl) was added to each well (*n* = 3) of a 96-well plate. Then, Folin-Ciocalteu reagent (100 μl) and 7% (w/v) Na_2_CO_3_ (80 μl) were added. The reaction mixture was incubated for 30 min at room temperature after thorough mixing. Finally, the absorbance was measured at 760 nm. With reference to the calibration curve of GA, the total phenolic content was calculated and is expressed in units of mg GA equivalents (Eq)/mg of extract.

### Analysis of caffeic acid content

The caffeic acid content in aqueous TL extract was determined using high-performance liquid chromatography (HPLC). Before analysis, the TL extract (25 mg) was dissolved in 2 ml water, filtered (a 0.45 μm filter), and diluted to various concentrations as a working solution. HPLC-UV analysis was performed using a Thermo Scientific Dionex Ultimate 3000 model (Thermo Scientific, MA, USA), which consisted of a variable wavelength detector (VWD-3100), an autosampler (WPS-3000SL), a tertiary pump (LPG-4300SD), and a column compartment (TCC-3000SL). Sample solution or caffeic acid standard (10 μl) was injected into a C18 analytical column (VertiSep™ HPLC columns, 250 mm × 4.6 mm, 5 μm particle size; Vertical Chromatography Co., Ltd., Nontaburi, Thailand). The HPLC conditions modified from those used in the previous study [16]. The mobile phases were composed of 60% (v/v) acetonitrile in water (solvent B) and 1% (v/v) acetic acid in water (solvent A). The flow rate was 0.7 ml/min, and the column was eluted with a linear gradient program of 30–40% solvent B over 0–7 min, 40–70% solvent B over 7–10 min, and 70–100% B over 10–28 min. The mobile phase was returned to 30% solvent B and maintained for 3 min to equilibrate the column before the next injection. The column compartment temperature was set to 30 °C, and the eluted compounds were monitored at 330 nm. The column pressure was not precisely controlled during HPLC analysis. Instead, it was varied according to the gradient compositions of the mobile phase when flow rate of mobile phase was fixed. The analytical system operates at a pressure of between 100 and 140 bar, approximately. In response to the increased concentration of acetonitrile in the mobile phase, the pressure is reduced. The analysis was performed in triplicate.

### Experimental animals and treatments

The study was performed in a manner of reduced the animal numbers and minimized animal suffering. Sixteen male Wistar rats (*Rattus norvegicus*) aged 6 weeks were obtained from Nomura Siam International Co, Ltd. (Bangkok, Thailand). The rats were maintained under constant temperature (23 ± 2 °C) and relative humidity (50–60%) with a 12 h light/dark cycle. Food and water were provided ad libitum. The animal experimental procedures were performed in a way that adhered to the Animal Research: Reporting of In Vivo Experiments (ARRIVE) guidelines.

The rats were randomly divided into 4 groups (4 rats per group): the control group, in which rats received subcutaneous injection of 1 ml/kg body weight (BW) normal saline once a week for 6 weeks; the PQ group, in which rats received subcutaneous injection of 18 mg/kg BW PQ dichloride once a week for 6 weeks; the PQ + TL-LD group, in which rats received subcutaneous injection of 18 mg/kg BW PQ dichloride and oral gavage of low-dose TL leaf extract (100 mg/kg BW) once a week for 6 weeks; and the PQ + TL-HD group, in which rats received subcutaneous injection of 18 mg/kg BW PQ dichloride and oral gavage of high-dose TL leaf extract (200 mg/kg BW) once a week for 6 weeks. PQ and TL treatment was performed according to the procedures of Orito et al. [17] and Tangpong and Satarug [10]. The rats were euthanized by thiopental sodium overdose (100 mg/kg BW) anesthesia. The abdominal cavities were then opened, and the lungs were excised.

### Determination of malondialdehyde (MDA) levels

MDA measurement was carried out using an OxiSelect™ TBARS Assay Kit (cat. no. STA-330, Cell Biolabs, Inc., USA) in accordance with the manufacturer’s protocol. Lung homogenate (50 mg/ml) was prepared by homogenizing lung sections on ice in phosphate-buffered saline containing 1× butylated hydroxytoluene. The homogenate was centrifuged at 10,000×*g* for 5 min, and the MDA content was assayed [18].

### Histopathology

Lung tissues were fixed in a 10% neutral buffered formalin solution, processed, and embedded in paraffin. The tissues were sliced into sections using a rotary microtome and subjected to hematoxylin and eosin (H & E) staining. The severity of pathological alterations was graded using the following semiquantitative scale: 0 = normal, 1 = pathological alterations in up to 25% of the high-power field, 2 = pathological alterations in 26–50% of the high-power field, 3 = pathological alterations in 51–75% of the high-power field, and 4 = pathological alterations in > 75% of the high-power field.

### Determination of pulmonary NOX, IL-1β and TNF-α mRNA expression using reverse transcription-polymerase chain reaction (RT-PCR)

Total RNA was extracted from lung tissue using a Tissue Total RNA Mini Kit (Geneaid, Korea). The purity and quantity of RNA were determined using a NanoDrop™ one/one^C^ Microvolume UV-Vis Spectrophotometer with Wi-Fi (Thermo Scientific, USA). RT-PCR was performed to amplify the genes. The thermal cycling conditions included an initial denaturation step at 95 °C for 15 min, denaturation at 94 °C for 1 min, primer annealing at 65 °C for 1 min, extension at 72 °C for 1 min, and a final elongation step at 72 °C for 10 min. The primers used are shown in Table 1. The DNA samples were loaded into a 2% agarose gel. After staining with ethidium bromide, the gel was visualized with a UV transilluminator. The amount of PCR product was detected using GeneTools software via image analysis (Syngene, Frederick, MD, USA).

**Table 1:** Primers of NOX, IL-1β, TNF-α and β-actin

Gene	5′-3′ Primer sequence	
NOX [19]	Forward primer	GGAAATAGAAAGTTGACTGGCCC
	Reverse primer	GTATGAGTGCCATCCAGAGCAG
IL-1β [20]	Forward primer	CCCTGCAGCTGGAGAGTGTGG
	Reverse primer	TGTGCTCTGCTTGAGAGGTGCT
TNF-α [20]	Forward primer Reverse primer	GACCCTCACACTCAGATCATCTTCT TGCTACGACGTGGGCTACG
β-Actin [21]	Forward primer	TTCTTTGCAGCTCCTTCGTTGCCG
	Reverse primer	TGGATGGCTACGTACATGGCTGGG

### Immunohistochemistry of pulmonary IL-1β and TNF-α

Lung sections were deparaffinized, rehydrated, and heated in sodium citrate buffer solution at pH 6.0 (Merck, Germany) using a microwave. Endogenous peroxidase activity was blocked in 3% H_2_O_2_ in distilled water. The sections were incubated with blocking buffer (normal goat serum) at room temperature for 30 min to block nonspecific binding sites. They were then incubated with an optimized primary antibody solution containing rabbit anti-mouse IL-1β and TNF-α antibodies (Abcam, Cambridge, MA, USA) in a humidified chamber at 4 °C overnight before being incubated with appropriately diluted secondary antibodies in a humidified chamber at room temperature for 30 min. An avidin-biotin complex (VECTASTAIN ABC Kit, Vector Laboratories, USA) conjugated with horseradish peroxidase was added to the sections, and diaminobenzidine (DAB; Vector Laboratories, USA) was applied for 3 min. After counterstaining with Mayer’s hematoxylin (Merck, Germany), the sections were dehydrated and mounted. All slides were randomly scored in 50 microscopic fields at high magnification as follows: 0 = no immunopositive cells; 1 = 1–25% immunopositive cells; 2 = 26–50% immunopositive cells; 3 = 51–75% immunopositive cells; and 4 = > 75% immunopositive cells [22].

### Statistical analysis

The results are expressed as the mean ± standard error of the mean (SEM). Differences between groups were determined using one-way analysis of variance. Post hoc testing was performed for group comparisons using the least significant difference test. Values of *p* < 0.05 were considered to indicate significance.

## Results

### Aqueous TL leaf extract contained total phenolic compounds and caffeic acid

Based on the Folin-Ciocalteu colorimetric method, linear regression was performed between the GA concentrations and the absorbance (y = 0.0039x + 0.0414, “R2” = 0.9996). The phenolic content of the extract was 20.1 ± 0.39 μg GA Eq/mg extract. The linearity of HPLC-UV determination was in the concentration range of 1.56–100 μg/ml (“R2” = 0.9999). Within the range of analysis, the coefficient of variation was less than 3%. The caffeic acid content in the extract was 0.31 ± 0.01 μg/mg.

### Aqueous TL leaf extract alleviated PQ-induced increases in MDA levels in lung tissue

**Fig. 1:**
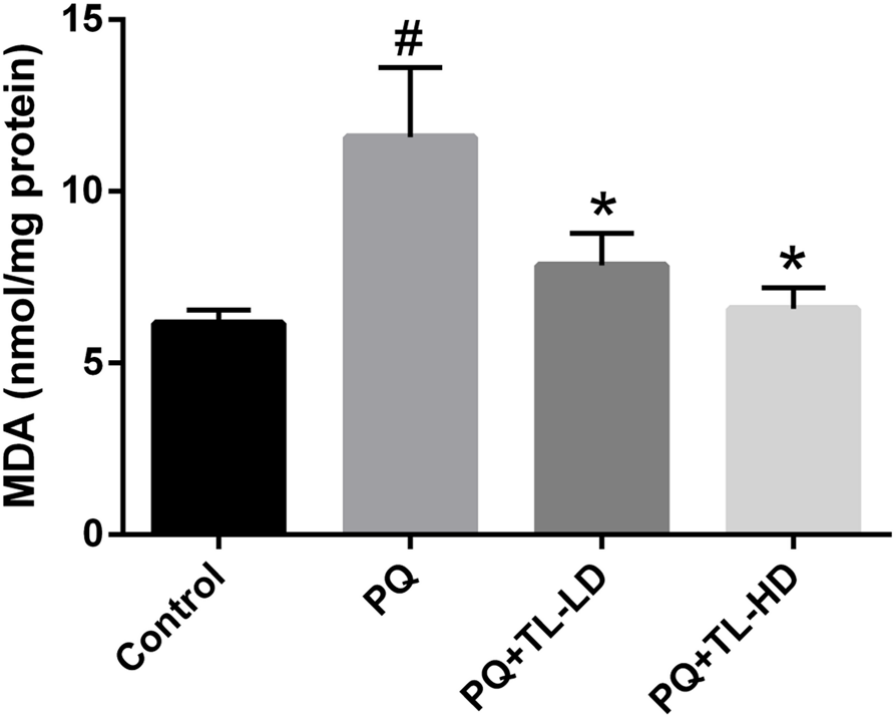
Pulmonary MDA levels of the study groups. The results are expressed as the means ± SEMs (*n* = 4 per group). ^#^*p* < 0.05 compared with the control group. ^*^p < 0.05 compared with the PQ group

As shown in Fig. 1, we evaluated the antioxidant effects of aqueous TL leaf extract against PQ-induced oxidative damage in male Wistar rats by detecting the levels of MDA, a biomarker of lipid peroxidation, using an assay kit. We found that the PQ group showed significantly higher MDA levels than the control group (*p* < 0.05). However, both the PQ + TL-LD group and the PQ + TL-HD group exhibited significantly lower MDA levels than the PQ group (*p* < 0.05).

### Aqueous TL leaf extract improved PQ-induced pathological alterations in lung tissue

**Fig. 2:**
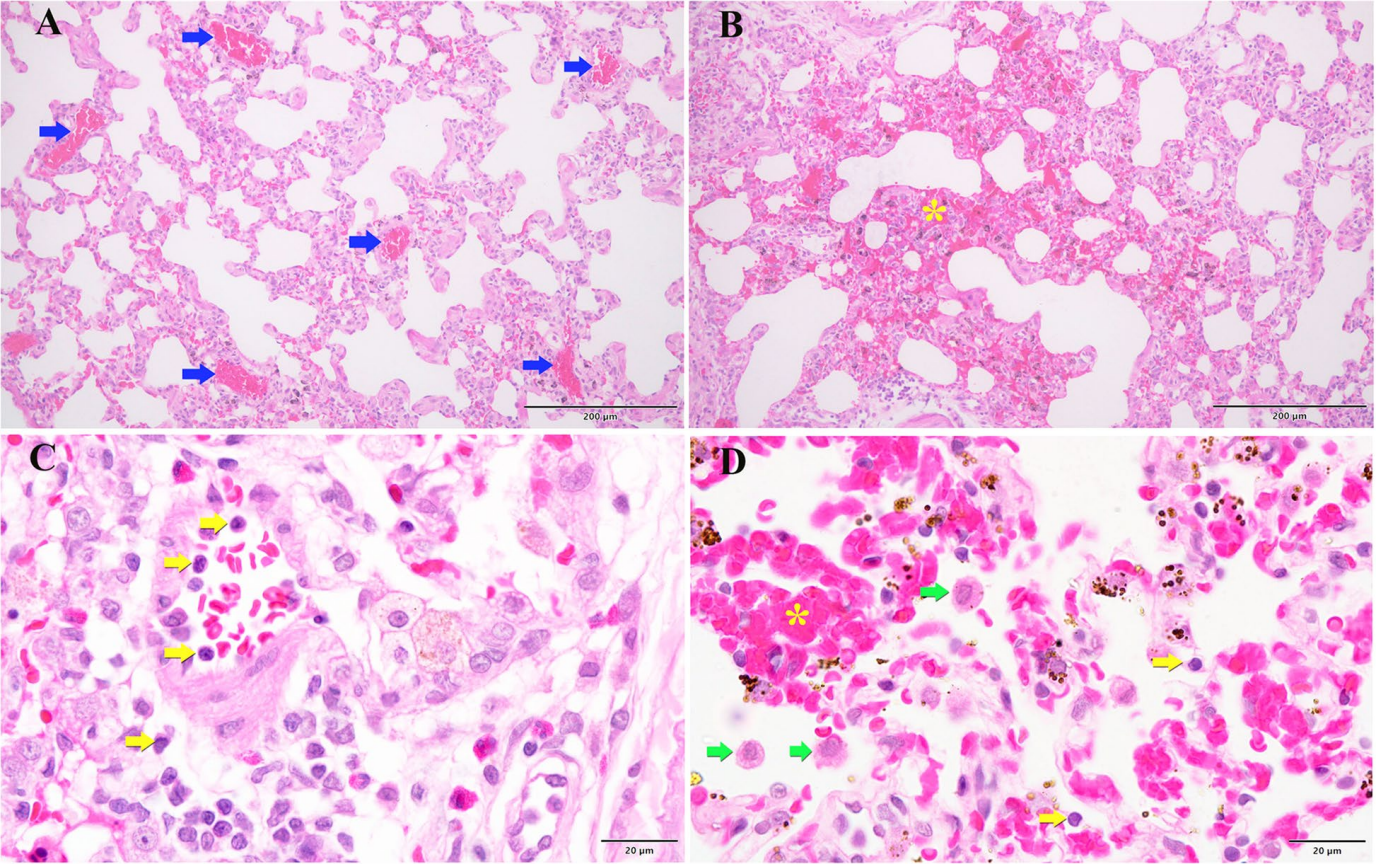
Lung histopathology of male Wistar rats (H&E staining) in the PQ group. The blue, yellow and green arrows indicate vascular congestion, lymphocytes and alveolar macrophages, respectively. The yellow asterisks indicate hemorrhage. Scale bar = 200 μm (A and B) or 20 μm (C and D)

**Fig. 3:**
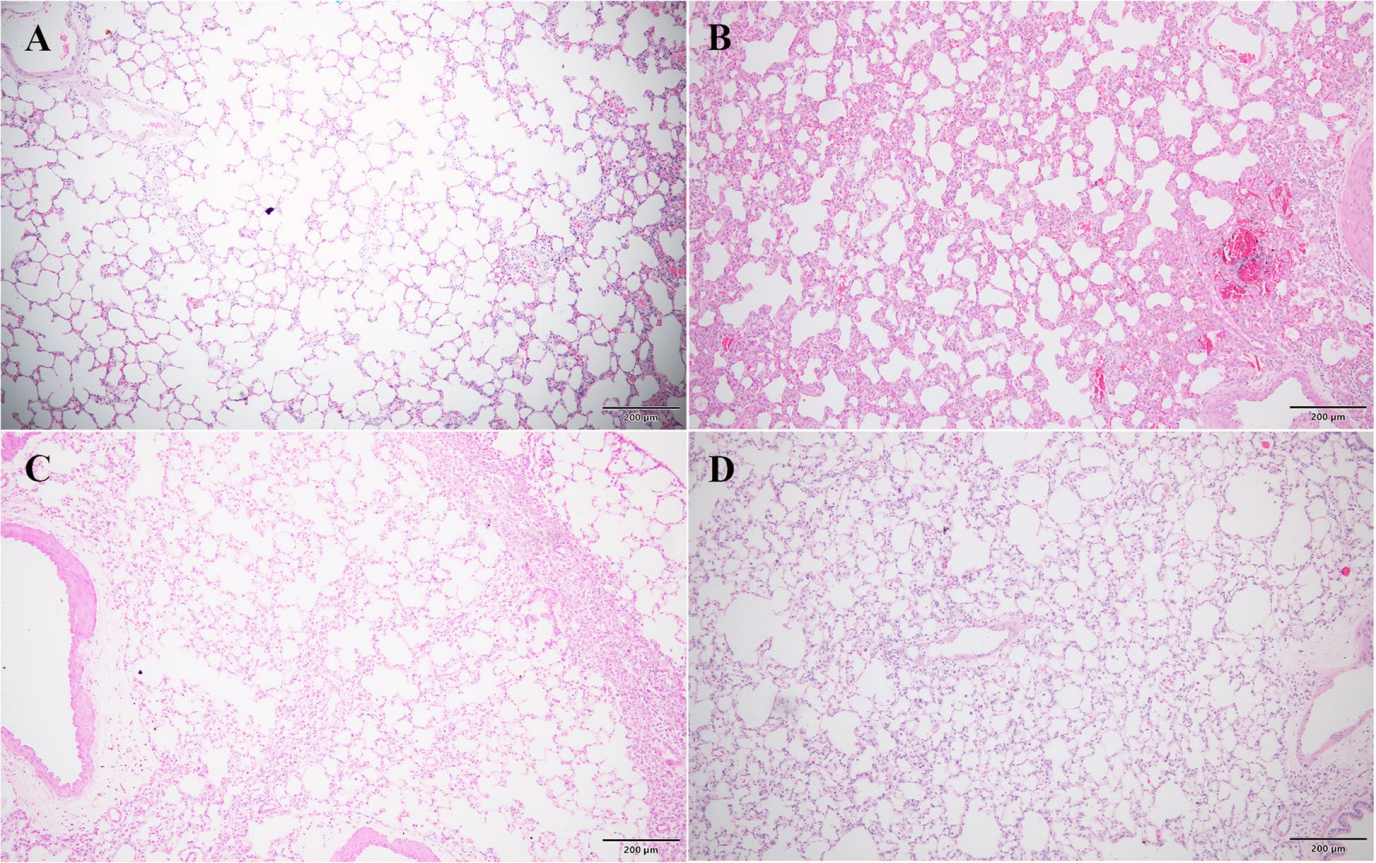
Lung histopathology of male Wistar rats (H&E staining) in the control (A), PQ (B), PQ + TL-LD (C) and PQ + TL-HD (D) groups. Scale bar = 200 μm

As shown in Figs. 2 and 3, the PQ group had significantly higher severity scores than the control group for pathological changes including alveolar edema, diffuse alveolar collapse, hemorrhage, leukocyte infiltration, alveolar septal thickening and vascular congestion (*p* < 0.001). The PQ + TL-LD group exhibited significantly less alveolar edema, diffuse alveolar collapse and hemorrhage than the PQ group (*p* < 0.05). Moreover, the PQ + TL-HD group demonstrated significantly less alveolar edema, diffuse alveolar collapse and hemorrhage, leukocyte infiltration, alveolar septal thickening and vascular congestion than the PQ group (*p* < 0.05) (Table 2).

**Table 2:** Lung pathological scores in the study groups

Pathology	Group			
	Control	PQ	PQ + TL-LD	PQ + TL-HD
Alveolar edema	0	2.38 ± 0.24^##^	1.38 ± 0.38^*^	0.81 ± 0.24^*^
Diffuse alveolar collapse	0	2.63 ± 0.38^##^	1.69 ± 0.24^*^	1.06 ± 0.26^*^
Hemorrhage	0	2.56 ± 0.30^##^	1.81 ± 0.3^*^	1.31 ± 0.19^*^
Leukocyte infiltration	0	2.31 ± 0.37^##^	1.75 ± 0.34	1.19 ± 0.28^*^
Alveolar septal thickening	0	2.44 ± 0.26^##^	1.88 ± 0.22	1.31 ± 0.19^*^
Vascular congestion	0	2.50 ± 0.29^##^	1.63 ± 0.39	1.06 ± 0.36^*^

The results are expressed as the means ± SEMs (*n* = 4 per group). ##*p* < 0.001 compared with the control group. *p < 0.05 compared with the PQ group

### Aqueous TL leaf extract downregulated the expression of NOX, IL-1β and TNF-α in the lung tissues of PQ-treated rats

**Fig. 4:**
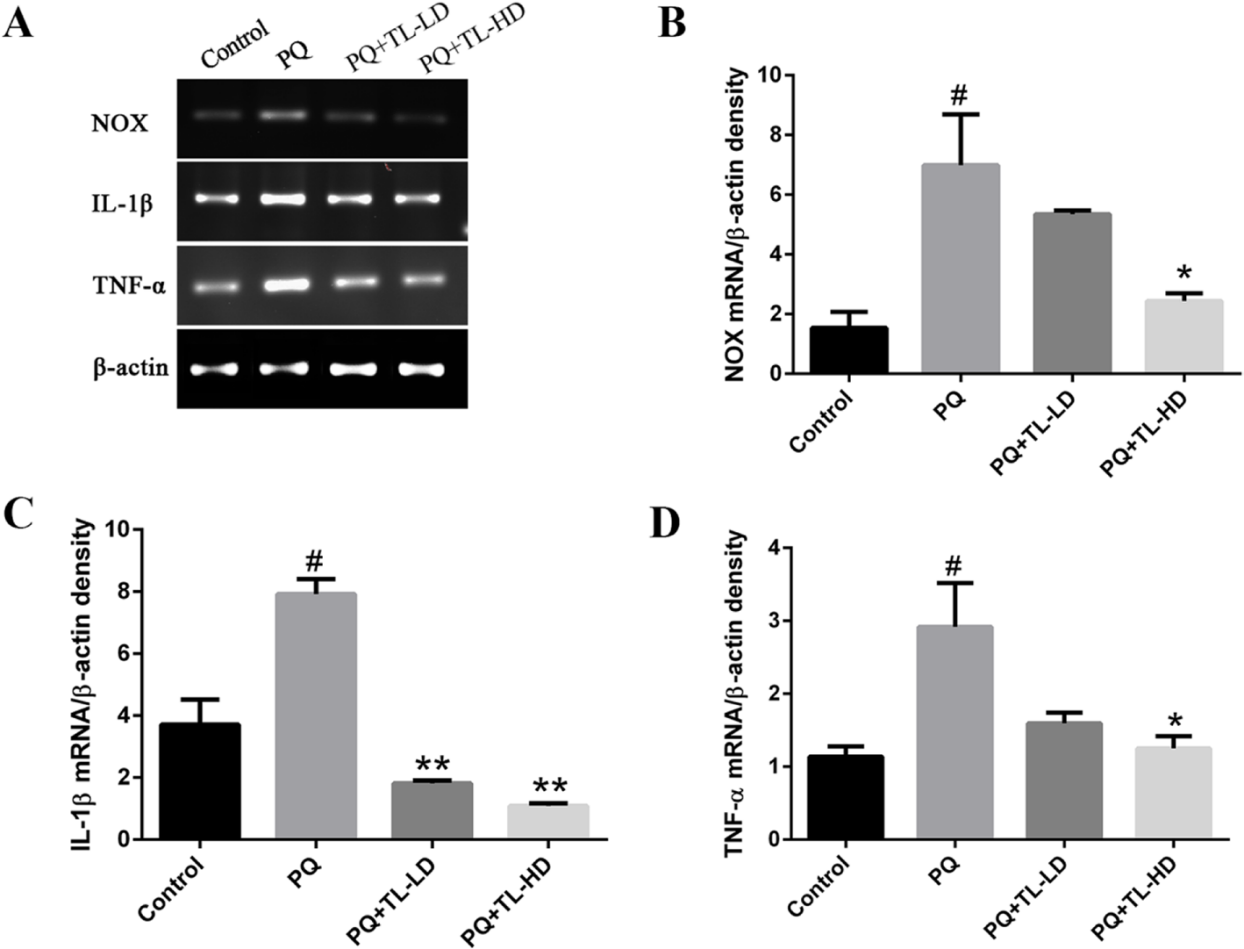
The mRNA expression (A) and mRNA levels of NOX (B), IL-1β (C) and TNF-α (D) in the study groups. The results are expressed as the means ± SEMs (*n* = 4 per group). ^#^*p* < 0.05 compared with the control group. *p < 0.05, **p < 0.001 compared with the PQ group

As illustrated in Fig. 4, Supplementary File 1 and 2, we evaluated anti-inflammatory ability by detecting the mRNA expression of NOX, IL-1β and TNF-α in the lung tissues of PQ-treated rats using RT-PCR. The results showed that the PQ group had significantly higher mRNA expression of NOX, IL-1β and TNF-α than the control group (*p* < 0.05). However, both the PQ + TL-LD group and the PQ + TL-HD group exhibited significantly lower mRNA expression of IL-1β (*p* < 0.001) than the PQ group, and the PQ + TL-HD group exhibited significantly lower mRNA expression of NOX (*p* < 0.05) and TNF-α (*p* < 0.05) than the PQ group.

### Aqueous TL leaf extract reduced the expression of IL-1β and TNF-α in the lung tissues of PQ-treated rats

**Fig. 5:**
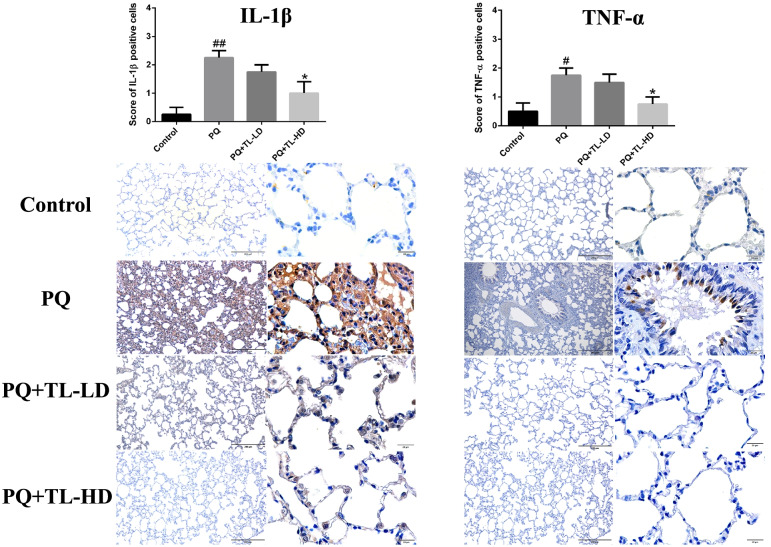
Immunohistochemistry of pulmonary IL-1β and TNF-α in male Wistar rats (*n* = 4 per group). ^#^*p* < 0.05 compared with the control group. *p < 0.05 compared with the PQ group

Immunohistochemistry showed that the PQ group had significantly higher expression of IL-1β and TNF-α than the control group (*p* < 0.001 and *p* < 0.05, respectively). However, the PQ + TL-HD group exhibited significantly lower expression of IL-1β and TNF-α than the PQ group (*p* < 0.05) (Fig. 5).

## Discussion

PQ-induced lung pathology has been thoroughly examined in both humans and experimental animals. The severity of PQ poisoning has been reported to exhibit dose and time dependence [23]. This study demonstrated the occurrence of PQ toxicity in the lungs of experimental rats that were administered 18 mg/kg BW PQ by subcutaneous injection once a week for 6 weeks. The pathological changes included alveolar edema, diffuse alveolar collapse, hemorrhage, leukocyte infiltration, alveolar septal thickening and vascular congestion. The primary targets of PQ are type I and II pneumocytes and Clara cells, which accumulate PQ via the polyamine transport system [24]. In type I alveolar cells, PQ causes swelling followed by vacuolation and disruption of organelles [4]. In type II alveolar cells, PQ induces the apoptosis of human lung type II alveolar epithelial cells via the ER stress pathway in vitro [25]. Loss of type II pneumocytes affects the synthesis and secretion of surfactant, eventually resulting in increased intra-alveolar surface tension, alveolar edema and collapse [26, 27]. PQ poisoning also causes pulmonary hemorrhage as a result of increased capillary endothelial permeability and vasoconstriction of the respiratory bronchiolar arterioles [3, 28].

Oxidative stress results from imbalance between the production and clearance of oxidants, including ROS and reactive nitrogen species, which can damage lipids, proteins and DNA [29]. Recently, studies on oxidative stress have widely used biomarkers such as MDA, an indicator of lipid peroxidation, to measure oxidative stress status [30]. Generally, the lipid peroxidation process comprises three steps, initiation, propagation and termination; during this process, oxidants attack lipids containing carbon-carbon double bonds, eventually producing lipid peroxidation products [31]. In this study, PQ treatment increased the levels of pulmonary MDA, indicating that the pathogenesis of PQ-induced lung damage is associated with ROS generation and oxidative stress [32]. ROS play a crucial role in oxidative stress upon PQ exposure, leading to the development of PQ-induced lung injury [6]. ROS are byproducts of aerobic metabolism and include mainly O_2_^•-^, ^•^OH and H_2_O_2_. ROS are produced within cells and organelles, such as mitochondria, peroxisomes, the endoplasmic reticulum and the plasma membrane [33]. O_2_^•-^, the most common ROS, is produced by transfer of an electron to molecular oxygen. Enzymes including xanthine oxidase, lipoxygenase, cyclooxygenase and NOX have been reported to be potential sources of O_2_^•-^ [33]. In this study, PQ increased the expression of NOX mRNA. The results indicated that PQ can induce ROS generation and oxidative stress in the lungs by upregulating NOX. Notably, PQ has been reported to stimulate leukocyte infiltration into the interstitial and alveolar spaces and to increase the production of pro-inflammatory cytokines, such as IL-6, TNF-α and IL-1β1 [34]. This study demonstrated that PQ upregulated the mRNA expression of the pro- inflammatory cytokines IL-1β and TNF-α, indicating that inflammation plays a crucial role in the mechanism by which PQ induces lung injury.

TL is a traditional Thai medicine belonging to the Acanthaceae family that is used as a therapeutic agent for alcohol and drug addiction [14]. Aqueous TL leaf extract has been reported to possess antioxidant, antidiabetic, anti-inflammatory, and antipyretic properties [11]. The anti-oxidative stress effects of aqueous TL leaf extract have been shown both in vitro and in vivo [35, 36]. The results of this study showed that aqueous TL leaf extract reduced the mRNA expression of NOX and reduced the levels of pulmonary MDA in rats treated with PQ. We suggest that antioxidation via inhibition of NOX may be the key mechanism by which aqueous TL leaf extract attenuates PQ-induced oxidative stress in the lungs. In addition, in this study, aqueous TL leaf extract contained total phenolic compounds and caffeic acid, which have been shown to play key roles in antioxidant defense [37]. This extract has also been reported to have anti-inflammatory effects [38]. Moreover, our findings demonstrated that aqueous TL leaf extract downregulated IL-1β and TNF-α mRNA expressions in the lungs of rats treated with PQ, indicating that the extract induced anti-inflammatory effects to ameliorate PQ-induced lung injury. Surprisingly, this extract, especially at a high dose, attenuated pathological alterations in the lungs induced by PQ. We suggest that aqueous TL leaf extract can ameliorate PQ-induced lung pathology by alleviating oxidative damage and inflammation.

## Conclusion

This study indicates that PQ causes oxidative stress and inflammation in male Wistar rats, leading to pathological alterations in the lungs. In addition, the results reveal that aqueous TL leaf extract can ameliorate PQ-induced lung pathology by alleviating oxidative stress via inhibition of the expression of NOX and by attenuating inflammation via inhibition of the expression of the pro-inflammatory cytokines IL-1β and TNF-α. We propose that active compounds in this extract, particularly phenolic compounds and caffeic acid, may exert crucial protective effects against PQ-induced lung injury. Thus, this study suggests that aqueous TL leaf extract can be used as an antidote for PQ-induced lung injury.

## Supplementary Information

**Additional file 1.**

**Additional file 2.**

## Data Availability

All data generated or analysed during this study are included in this published article and its supplementary information file.

## Abbreviations

BW: Body weight
FW: Forward primer
H & E: Hematoxylin and eosin
H_2_O_2_: Hydrogen peroxide
HPLC: High-performance liquid chromatography
IL-1β: Interleukin 1 beta
MDA: Malondialdehyde
NOX: NADPH oxidase
O_2_^•-^: Superoxide radical
OH: Hydroxyl radical
PQ: Paraquat
ROS: Reactive oxygen species
RT-PCR: Reverse transcription-polymerase chain reaction
SEM: Standard error of the mean
TL: *Thunbergia laurifolia*
TNF-α: Tumor necrosis factor alpha
