# Supplementary figures and images for: Aqueous Thunbergia laurifolia leaf extract alleviates paraquat-induced lung injury in rats by inhibiting oxidative stress and inflammation
Source: BMC Complement Med Ther. 2022 Mar 22;22:83. doi: 10.1186/s12906-022-03567-4 (PMC8939148; doi:10.1186/s12906-022-03567-4)

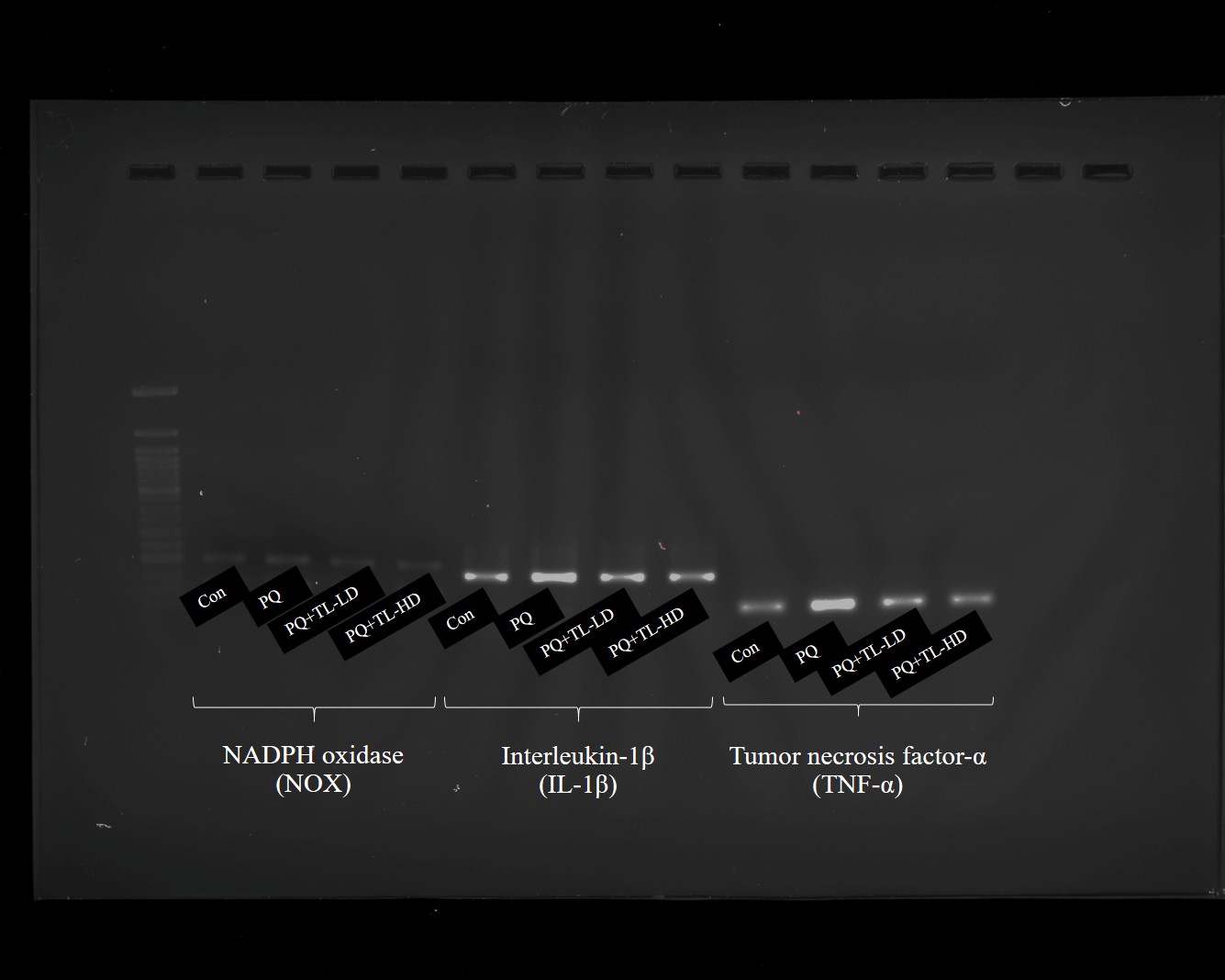

Supplement: Supplementary file 1 — Additional file 1. [file 12906_2022_3567_MOESM1_ESM.jpg]

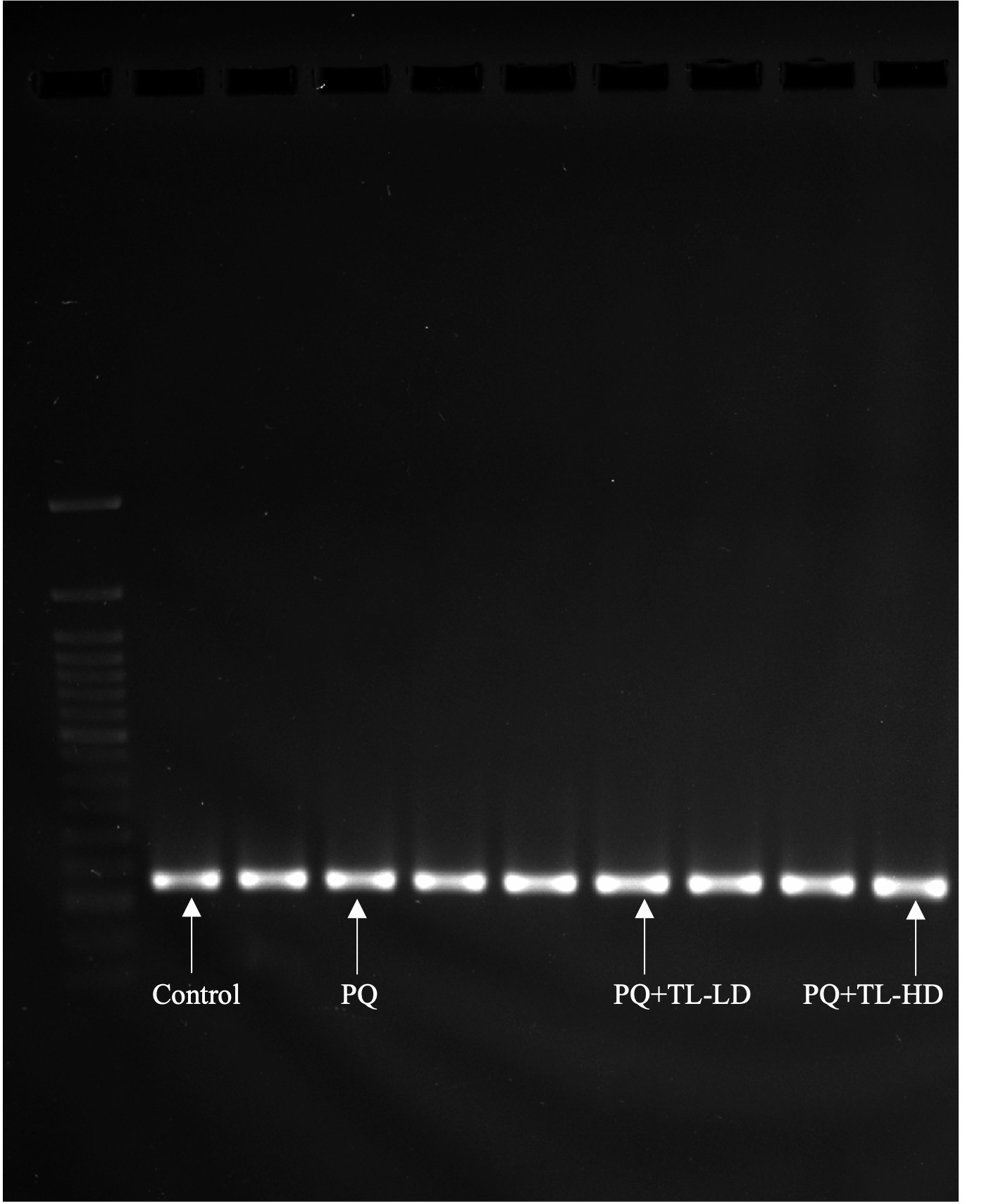

Supplement: Supplementary file 2 — Additional file 2. [file 12906_2022_3567_MOESM2_ESM.jpg]
